# From Boolean Network Model to Continuous Model Helps in Design of Functional Circuits

**DOI:** 10.1371/journal.pone.0128630

**Published:** 2015-06-10

**Authors:** Bin Shao, Xiang Liu, Dongliang Zhang, Jiayi Wu, Qi Ouyang

**Affiliations:** 1 The State Key Laboratory for Artificial Microstructures and Mesoscopic Physics, School of Physics, Peking University, Beijing, China; 2 The Center for Quantitative Biology and Peking-Tsinghua Center for Life Sciences, Peking University, Beijing, China; University of Georgia, UNITED STATES

## Abstract

Computational circuit design with desired functions in a living cell is a challenging task in synthetic biology. To achieve this task, numerous methods that either focus on small scale networks or use evolutionary algorithms have been developed. Here, we propose a two-step approach to facilitate the design of functional circuits. In the first step, the search space of possible topologies for target functions is reduced by reverse engineering using a Boolean network model. In the second step, continuous simulation is applied to evaluate the performance of these topologies. We demonstrate the usefulness of this method by designing an example biological function: the SOS response of *E*. *coli*. Our numerical results show that the desired function can be faithfully reproduced by candidate networks with different parameters and initial conditions. Possible circuits are ranked according to their robustness against perturbations in parameter and gene expressions. The biological network is among the candidate networks, yet novel designs can be generated. Our method provides a scalable way to design robust circuits that can achieve complex functions, and makes it possible to uncover design principles of biological networks.

## Introduction

Synthetic biology is an emerging field focusing on understanding the behaviors of biological systems through designing and constructing of synthetic gene circuits. Inspired by electric circuits and cellular systems, various synthetic genetic circuits have been created, including toggle switch [1], oscillator [2, 3], counter [4], and logic gates [5]. In addition, multiple well-characterized parts are combined together to achieve more complex functions, such as biosensing [6], edge detection [7] and Pavlovian-like conditioning [8]. However, designing circuits with complex functions remains a challenge. In previous work, methods based on continuous simulation have been developed to select out networks that are capable of executing different functions. A very useful method is enumeration of network structures [9–11]. Through computer simulation, the full range of possible network architecture was explored and the interacting network of biological components was converted into a set of differential equations. Solutions to these ordinary differential equations (ODEs) provided the dynamics of each component of the network. Functional topologies with fewer parameter constraints can thus be selected. A list of these functions includes adaptation [9], dose-response alignment [10], and segment polarity in development [11]. However, enumeration of network structures usually leads to a dramatic rise in computational cost with increasing number of genes, thus making it difficult to scale-up. In other approaches, network structures are evolutionarily optimized from a finite set of independent circuits. However, this method may not result in an optimal design, as only a limited space of topology is sampled [12–14].

On the other end of the spectrum lies the class of discrete models ranging from simple logic circuits to finite state machines and Boolean networks. In these models, each component in the system has only two states: ON (1) and OFF (0). The regulation rule for each component is defined by Boolean functions, such as two-input AND gate and OR gate. In electronic circuit design, constructing digital circuits with a given truth table and logic gates is a standard procedure called combinational logic design. One of the basic methods is “Karnaugh map”, which is also applied in the design of biological digital circuits [15]. However, it is difficult to construct biological circuits with sequential logic behaviors using standard methods of electronics, because these methods usually involve flips-flops that are quite rare in standard biological parts [16]. Boolean network model has been used to illustrate the dynamic behaviors of biological systems and to reconstruct biological networks underlying specific functions, thus contributing to a natural method of constructing sequential logic circuits in biology [17, 18]. Although these discrete models are quite efficient in computation, they suffer from several inherent limitations: It is unclear whether the circuits designed using discrete framework can execute desired functions robustly in a wet-lab implementation. In addition, it is difficult to rank the possible circuits based on their ability to tolerate perturbations in parameters and gene expression levels.

**Fig 1:**
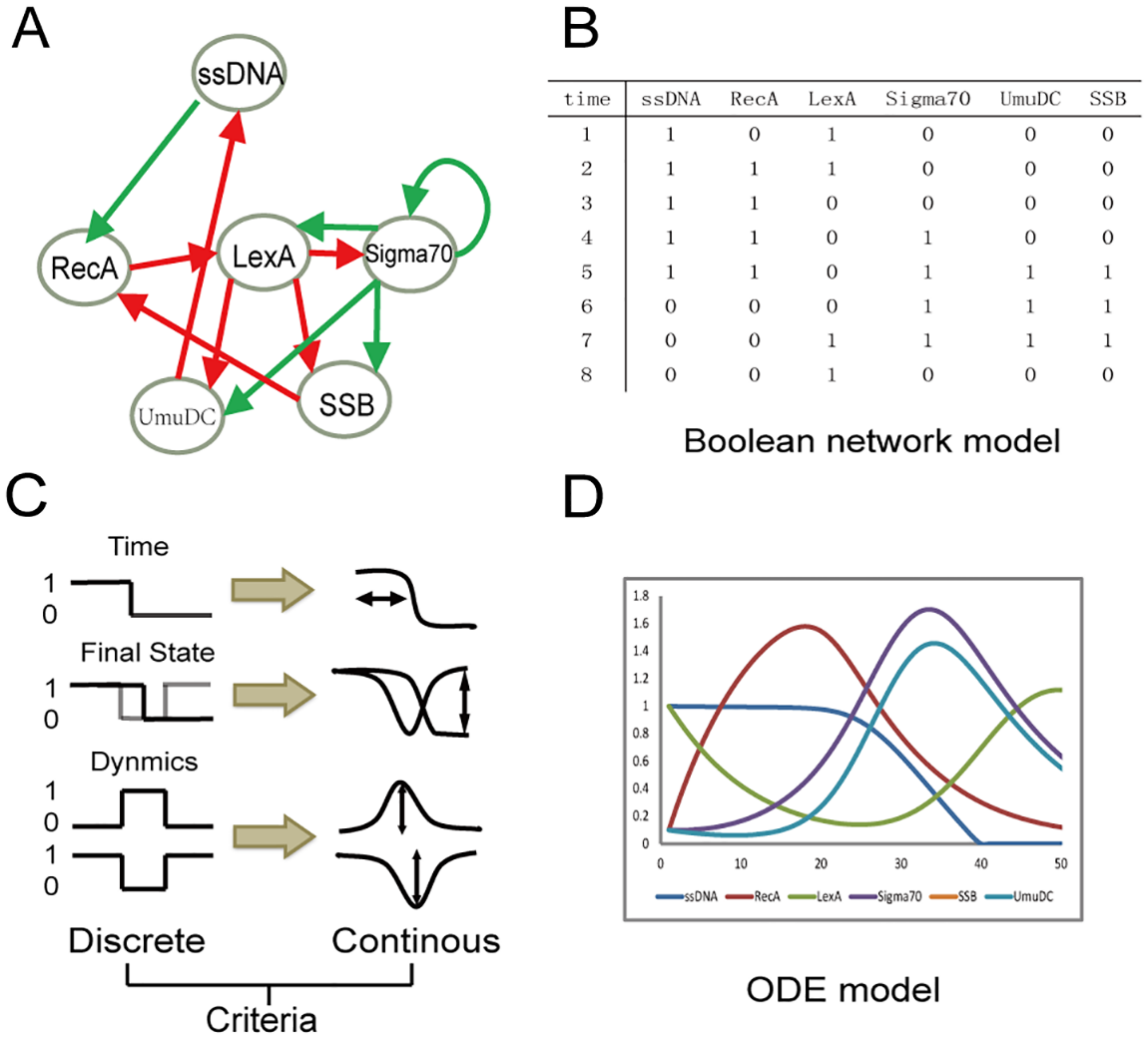
The SOS network and its dynamics. (A) Regulatory network of the SOS response of *E*. *coli*. The nodes represent the signal and the essential proteins. The green lines represent activation and the red lines represent inhibition. (B) Dynamics of the SOS response in the Boolean network model. (C) Three criteria and their representations in the discrete and continuous model. The first criteria addresses degradation time of ssDNA, i.e., ssDNA should be down-regulated to zero at the end of the simulation. In the second criteria, the final state of the system should return to the initial state, except for the degradation of ssDNA. The third criteria requires that the dynamics of each node in the ODE model should be in accordance with those in Boolean trajectory. (D) Example of a successful response in the ODE model.

In this paper, we present a two-step method that combines the discrete model and the continuous model to generate a novel design of functional circuits. In our approach, first, a Boolean network model is applied to generate candidate networks that are better capable of executing the target functions. Then, continuous simulation is used to quantitatively assess the robustness of these candidate networks. Here, we focus on one critical biological behavior, the SOS response in *E*. *coli*., wherein DNA repair is induced in response to the existence of a single stranded DNA (ssDNA). The desired function of this network is as follows: Upon accumulation of ssDNA, RecA is recruited to the single stranded regions of DNA and becomes activated. Activation of RecA releases the inhibition of SOS genes by facilitating self-cleavage of their repressor LexA. The main activator of SOS gene is σ^70^, which belongs to a family of transcription initiation factors responsible for stress response. Its downstream genes can be simplified into two genes, SSB and UmuDC, which are responsible for the repair of DNA damage and inhibition of RecA. When the DNA repair is completed, LexA is activated and the expression of SOS genes is down-regulated [19, 20]. The natural network performing this function is presented in Fig 1A. This DNA damage response may represent a large class of response pathways and we use our approach to design functional circuits underpinning this function. The analysis of functional circuits obtained by our approach allows us to discover core motifs responsible for robust response.

## Methods

### Boolean Network Model and Reverse Engineering

In the Boolean network model, each node represents a biological species. S_*i*_(t)∊{0,1} represents the state of node i at time t. The network topology can be described by its connection matrix A, in which *a*_ij_ indicates regulation from node i to node j. *a*_ij_ is positive for activation and negative for inhibition [17]. As changes in weight of inhibition regulation render the Boolean trajectory nearly unchanged [21], we take a dominant inhibition form of regulation in our Boolean network model, which means that inhibition has a much larger weight than activation, i.e., |*a*_*inh*_| ≫ |*a*_*act*_|. If the state of node j is 1 (On) at time t and one of its inhibitor is activated, then *S*_*i*_(*t* + 1) = 0 regardless of all the activation terms. This captures the combinatorial mechanism of transcription regulation, in which existence of an inhibitor can block transcription of the target gene. Although some limitations persist, our method is able to recover the regulatory network from different types of data in an efficient way [22]. The level of genes in the next time step is determined by the level of genes in the current time step by the following rule:

{Si(t+1)=θ(∑jSj(t)aji),∑jSj(t)aji≠0Si(t+1)=Si(t),∑jSj(t)aji=0 (1)

Where *θ*(x) is a Heaviside step function with *θ*(x) = 1 for *x* > 0 and *θ*(x) = 0 for *x* < 0. In our example of SOS response pathway, LexA is constitutively expressed to bind to the SOS box of the target genes, whereas σ^70^ is not stimulated in the normal state. RecA is expressed in normal conditions, yet it only becomes functional when forming filaments around single stranded DNA. Thus only LexA and ssDNA are 1 in the initial state and the dynamics (trajectory) of the network can be generated according to Fig 1A and using Eq 1 until the system returns to its normal state, as illustrated in Fig 1B. In the first step of our circuit design method, the purpose is to generate a set of networks that can perform this trajectory of the SOS network.

We apply reverse engineering methods [18] in the first step to limit the number of possible topologies. Reverse engineering presents a class of methods aiming to uncover biological regulatory networks based on experimental data. In our inhibition dominant Boolean network model, the constraint of network topology by the Boolean trajectory can be represented in an analytical manner [21].

$$s_{i}(t+1)=\left(\sum_{j\neq i}(s_{j}(t)\cdot g_{ji})+s_{i}(t)\cdot \bar{r_{ii}}+\bar{s_{i}(t)}\cdot g_{ii}\right)\cdot \prod_{j\neq i}\left(\bar{s_{j}(t)\cdot r_{ji}}\right)$$ (2)

In the equation, *g*_ij_ and *r*_ij_ are the Boolean variables corresponding to activation and inhibition from node i to node j, respectively. The OR gate is indicated by addition (‘+’ and ‘∑’), and used to combine all activation terms in the first bracket, whereas inhibition terms are linked by AND gate, which is represented by multiplication (‘•’ and ‘∏’). The bar in Eq 2 denotes the NOT logic gate. For each node in the pathway, the logical constraints (Eq 2) of different time steps are combined together to get all possible regulations for that node. This reverse engineering method generates 7.1×10^6^ possible networks using the biological trajectory of SOS response.

### Minimal Network Constraint

When the number of nodes is fixed, the complexity of practical implementation of our design depends largely on the number of edges. Circuits with fewer edges are more favored in a wet-lab implementation. Minimal networks have been proposed to contribute to the core motif responsible for the main functional response [21]. Moreover, our previous work indicates that biological networks may prefer to use minimal networks to fulfill their functions, providing evidence that the edge number is limited in the evolutionary process [22]. To apply minimal network constraint in our approach, we enumerate all possible regulations of each node and obtain the ones with the fewest edges. These regulations are then combined together to obtain 48 minimal networks. By definition of our target function, we exclude all networks with self-loop on the input node, i.e., ssDNA.

### Continuous Model Simulation

To investigate the continuous dynamics of our designed circuits, we modeled the selected networks via ordinary differential equations (ODEs) in the second step. In the simulation, we limited ourselves to transcriptional regulatory networks, which are quiet common in synthetic biological networks. Input node is ssDNA, and all other nodes are assumed to be transcriptional factors (TFs) that can bind to upstream sequences of the target genes. In our model, Hill functions with Hill coefficient 2 are used to model the activation terms. The inhibition terms of nodes take a multiplication form, except for the input node (ssDNA), implying that the inhibitors are independent of one another and can independently block the transcription of the target gene. Repair of ssDNA is modeled by sum of the Hill function terms in order to take the cooperative nature of the functional proteins into account [23]. Basal production and degradation is also introduced in the equations. The ordinary equations are as follows:

$$\left\{ \begin{array}{c} \frac{dx_{i}}{dt}=(\sum_{positive}W_{P}a_{j}\frac{x_{j}^{N}\delta_{ij}}{b_{j}+x_{j}^{N}})\prod_{negative}\frac{1}{c_{j}+W_{N}d_{j}x_{j}^{N}}+0.1-x_{i}\neq 1\\ \frac{dx_{1}}{dt}=-\sum_{negative}\frac{x_{j}^{N}}{c_{j}+x_{j}^{N}} \end{array}\right.$$ (3)

In this equation, *b* denotes the dissociation constant and *a* denotes the kinetic constant. *δ* represents leakage of the promoter. *Wp* and *Wn* are the two parameters determining the relative weight of the inhibition and activation terms. All nodes are numbered as in Fig 1 to avoid confusion in the following analysis, i.e., ssDNA as node 1, RecA as node 2, LexA as node 3, σ^70^ as node 4, UmuDC and SSB as nodes 5 and 6. The initial value of node 1 (ssDNA) and node 3 (LexA) is set to be 1.0, and the rest of the nodes are set to be 0.1.

To evaluate the performance of candidate networks, quantitative criteria should be employed. Although we start from a Boolean trajectory, it is meaningless to reproduce the same trajectory in ODE simulations, partly because of the lack of time scale in the Boolean network model and arbitrary ways of discretization. We assume that the core dynamics of the DNA damage response lies behind the exact Boolean trajectory and can be abstracted to several criteria. Three conditions are assigned to capture the main characteristic of a successful response:

- Level of ssDNA (node 1) should be down-regulated to zero at the end of simulation, which is exactly the function we need.
- The final state of the system will be same as the initial state, except for the decrease of node 1, i.e., the system relaxes to its normal state after a transient response to input signal (node 1). At the initial state, LexA (node 3) is ON in order to repress the downstream SOS genes, so that the level of node 3 should be larger than any other nodes in the end.
- The dynamics patterns of nodes in continuous simulation must be the same as those in the Boolean trajectory; the correspondence is presented in Fig 1C. We identify the dynamic patterns of each node, which are classified into three categories, namely, ‘peak’, ‘valley’ and ‘decline’ according to the ‘shape’ of their time trajectories. The dynamics of ‘peak’ should have a maximum level of expression and ‘valley’ should have a minimum level.

In criteria ii), we restricted the final state of the system to be the normal state of the cell, in which only LexA is activated. Many response pathways share similar characteristics, yet it is not always true. For example, activation of the mating pathway of budding yeast drives the cell to a different phenotype called ‘shmoo’. Then, different criteria for the final state should be chosen to take into consideration the Boolean trajectory and the biological interpretations of the biological pathway. In addition, we add a constraint that maximum expression of each gene should be larger than 0.1. These criteria correspond to the main characteristics of the Boolean trajectory, leaving details, such as specific time of activation, unconstrained.

### Performance of circuit

**Fig 2:**
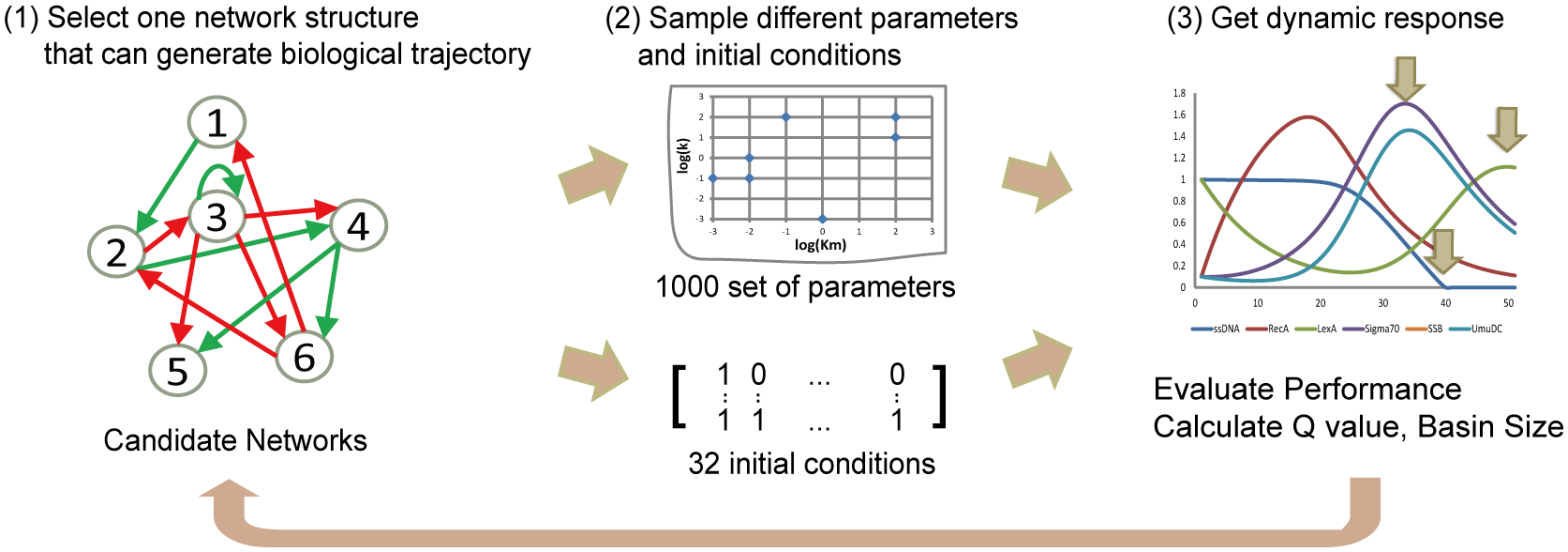
Workflow of continuous simulation. For networks selected by Boolean network model, 1000 random parameter sets and 32 initial conditions are used to quantitatively assess their robustness. The ordinary differential equations describing these transcriptional networks are solved numerically to generate the dynamics of each network component. The proportion of parameter sets and number of initial conditions that can achieve a successful response is termed as the Q value and basin size, respectively, for the corresponding network.

The evaluation process of circuit performance is presented in Fig 2, in which two scores that can reflect robustness of networks are employed. The first score is Q value that estimates the volume of the functional parameter space [9]. For each network, we randomly choose 1000 sets of parameters using Latin hypercube sampling. Starting from the same initial state, the network dynamics with each parameter set is obtained by solving the ODEs. Time series of all nodes are then checked to see if they present a successful response, i.e., if they meet three criteria above. For a particular network structure, the proportion of parameter sets that can achieve our desired function is defined as the Q value of that structure. A larger Q value implies that the network can generate successful response to DNA damage over a wide range of parameters and, hence, is less sensitive to parameter variation. The second score is relevant to robustness in state space. For the Boolean network model, the state of the system is updated using Eq 1 until it reaches a fixed point, which can also be called an attractor. The number of initial states that will flow into an attractor is defined as the basin size of that attractor. It is proposed that a biological state should have a large basin size in order to generate stability [17]. We held similar assumption in our design procedure that normal state with node3 activated should be a big attractor and stable against fluctuations in gene expression. In the Boolean network model, all possible initial conditions (2^*N*^ for network with N nodes) are enumerated to calculate the basin size, which is not practical in continuous simulation. To sample the space of initial states in the ODE model, we employ a similar approach in which the state of the nodes is treated as a continuous variable instead of a Boolean variable, e.g., the initial state (1.0, 0.1, 1.0, 0.1, 0.1, 1.0) is used instead of (1, 0, 1, 0, 0, 1). Criteria (i) and (ii) are used to see if the state of system flows into the normal state. Criteria (iii) is abandoned, as changing of the initial state may affect the dynamic patterns of the nodes. ssDNA is set to be 1.0, leaving 32 initial states in total.

## Results

### Performance of minimal networks and candidate networks

**Fig 3:**
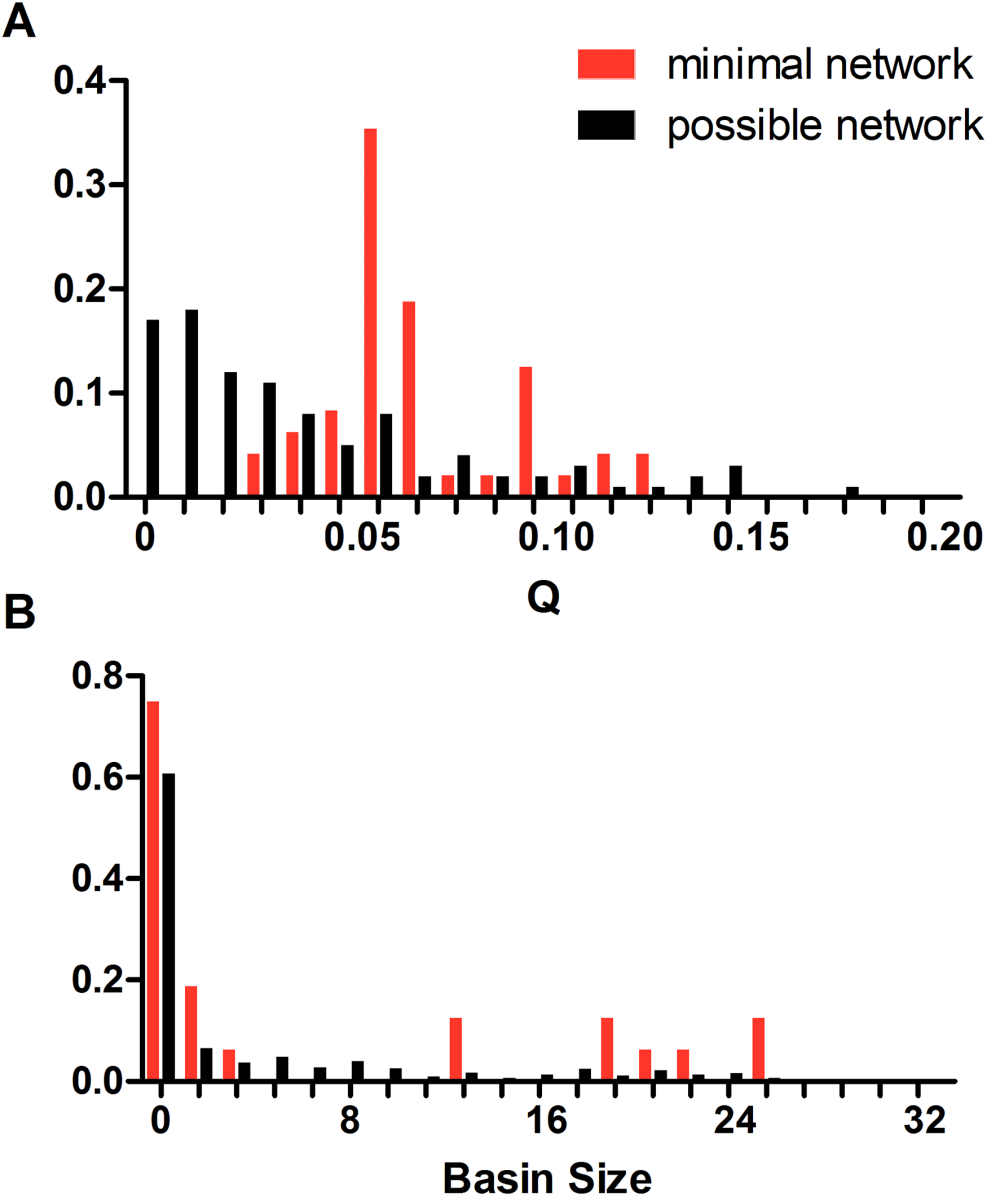
Distribution of Q value and basin size for minimal networks and candidate networks. (A) Distribution of Q value for minimal networks and candidate networks. Minimal networks are represented in red and the candidate networks in black. (B) Distribution of basin size for minimal networks and candidate networks.

In addition to minimal networks, we randomly selected 100 candidate networks that can generate the Boolean trajectory to compare the performance of both types of networks. The Q value distributions of minimal and candidate networks are illustrated in Fig 3A. Most of the first-step-selected networks have Q values larger than zero. On the contrary, only ~15% random networks have a positive Q value (data not shown), suggesting that filtering networks by the Boolean network model is efficient in obtaining the functional topologies. Former work has shown that the transform from differential equations to Boolean networks is possible under several assumptions [24]; our results reveal that the Boolean dynamics can also be transformed to continuous ones. Application of Boolean network model can largely reduce the search space of topologies in which most networks have a non-zero Q value, thus helping us focus on networks that are better capable of achieving target functions.

The distribution of Q value and basin size of minimal and candidate networks differs dramatically. For candidate networks, the proportion of networks decreases quickly as the Q value increases. Only a small fraction of networks can robustly execute the response function. Approximately 15% of the networks have a zero Q value, indicating that among all candidate networks filtered out in the first step, a small fraction is not functional in a more realistic circumstance. Although our criteria have relaxed the requirement for time sequence of events, they may be violated by some networks due to incoherence of regulations or mismatch of time scales. All minimal networks have a Q value larger than zero and the distribution peaks in 0.05, providing evidence that minimal networks are more robust against parameter perturbations. To discriminate between the two distributions in a quantitative way, we employed a measure of Jensen-Shannon Divergence (JSD), which describes the similarity of the two distributions and used statistical tests to examine the significance of difference. Our null hypothesis is that the distribution of minimal network and candidate networks have no significant difference, i.e., H_0_:JSD(p_*m*_,p_*c*_) = 0. The JSD of the Q value distribution for minimal networks and candidate networks is 0.31 with a p-value < 0.0001, indicating that the two distributions are significantly different. Similar results hold for the basin size distribution (Fig 3B), where the JSD of the two distributions is 0.13 with a p-value < 0.0001. Minimal networks are more likely to have a large basin size compared to candidate networks. Interestingly, although most networks we sampled have a full basin in the Boolean network model, only one of them has a full basin attractor in the continuous model simulation.

**Fig 4:**
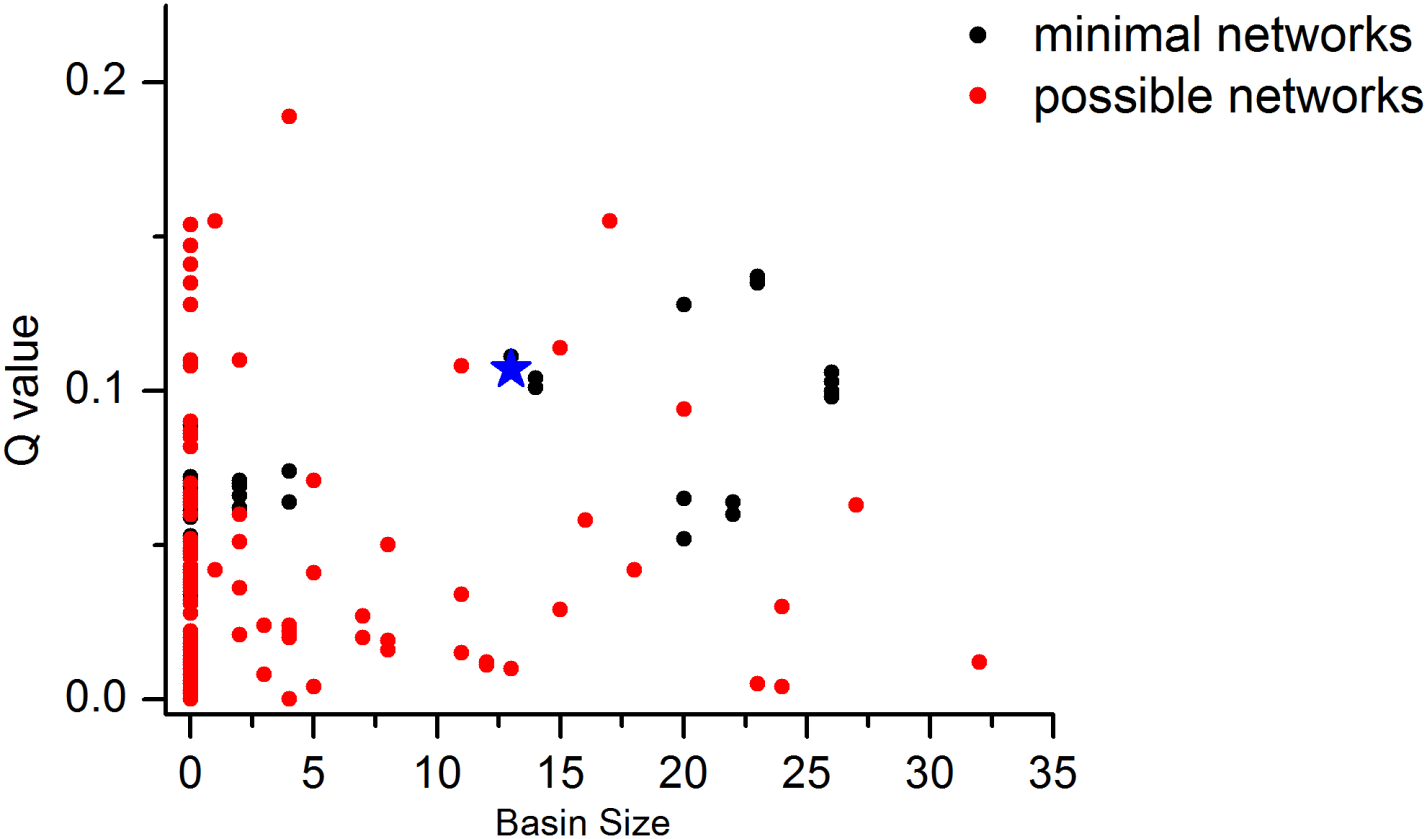
Performances of minimal networks and candidate networks. The distribution of Q value and basin size for minimal networks and candidate networks. Minimal networks are represented in red and candidate networks in black. The large blue star represents the biological network.

Our results suggest that minimal networks may be more robust to parameter and expression perturbations than candidate networks, which is apparent in Fig 4. Minimal networks tend to outperform the candidate networks, and mainly distribute in the upper right corner of the figure. In our previous work [22], we have proposed that least number of regulatory edges is preferred to implement biological functions. Our results here provide further evidence and suggest that minimal network constraints may also be useful in the design of functional circuits. As shown in Fig 4, the natural SOS network has a larger Q value and basin size than most networks, indicating that the biological design may be optimized in the course of evolution. Nonetheless, there are a small number of minimal networks that outperform the biological network; these networks may provide the basis for rational design of functional networks that can outperform the natural network.

### Topology analysis

**Fig 5:**
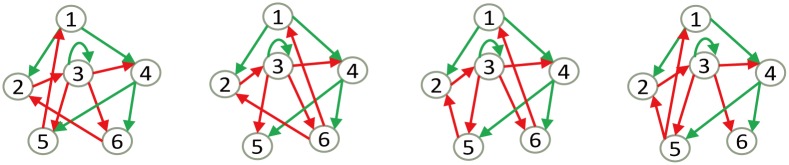
Core motifs responsible for successful response. Four minimal networks have larger Q values and basin sizes than the biological network. Aside from the edges shared by all of the candidate networks, all these topologies employed self-activation of node 3 and direct activation of node 4 by node 1, which contributes to the robustness of the network.

We next analyze the backbone motifs (minimal networks) responsible for robust response. Among all 48 minimal networks, the main difference is the activation mode of node 3 (LexA) and node 4 (σ^70^). In the biological network (Fig 1A), node 3 is activated by node 4, and node 4 has a self-activation loop, whereas 4 minimal networks that have a better performance than the biological network employ another regulation pattern (Fig 5). These networks all include self-activation of node 3, and activation of node 4 by node 1. The only difference is the different combination of inhibition of node 1 and node 2 by node 5 and node 6, which is not significantly different, as node 5 and node 6 both belong to the genes regulated by node 4. These results suggest that direct activation of node 4 by upstream signals may be more reliable than a self-activation loop in signal transduction. In addition, self-activation of node 3 may help build up its expression level when DNA is repaired.

## Discussion

By limiting the search space of networks through reverse engineering of the Boolean network model, we have developed an efficient method to generate networks that can achieve robust response to the DNA damage signal. Starting from a discretized time trajectory, reduction of candidate networks using Boolean network model can make the following simulation more practical. In a network of 6 interacting nodes, as in our case, there are 1.5×10^17^ different topologies, making it impossible to select out networks that are capable to perform the desired function by enumerating all topologies. Even when enumeration is possible, computational resources are largely wasted because only a small fraction of these networks are able to execute a successful response. Application of Boolean network model can reduce the number of possible networks by 10^10^ fold, and there only exist 48 minimal networks.

Networks found by the discrete model are better capable to reproduce the desired dynamics in continuous model simulation than random networks, which may suggest a good correspondence between the ODE and Boolean models in our problem. However, these networks are not equivalent in their ability to achieve robust response in more realistic implementation. Network performance is characterized by continuous model simulation under different parameters and initial conditions. The Q value and Basin size are used to quantitatively assess the ability of networks to robustly respond to the damage signal. Among all the candidate networks derived by the Boolean network model, those with the minimal number of edges are more robust than the rest. This implies that adding edges to minimal networks can barely improve the network performance. Biological networks usually not only have more edges, but also show better performance than minimal networks, providing evidence of the efficiency of network evolution.

**Fig 6:**
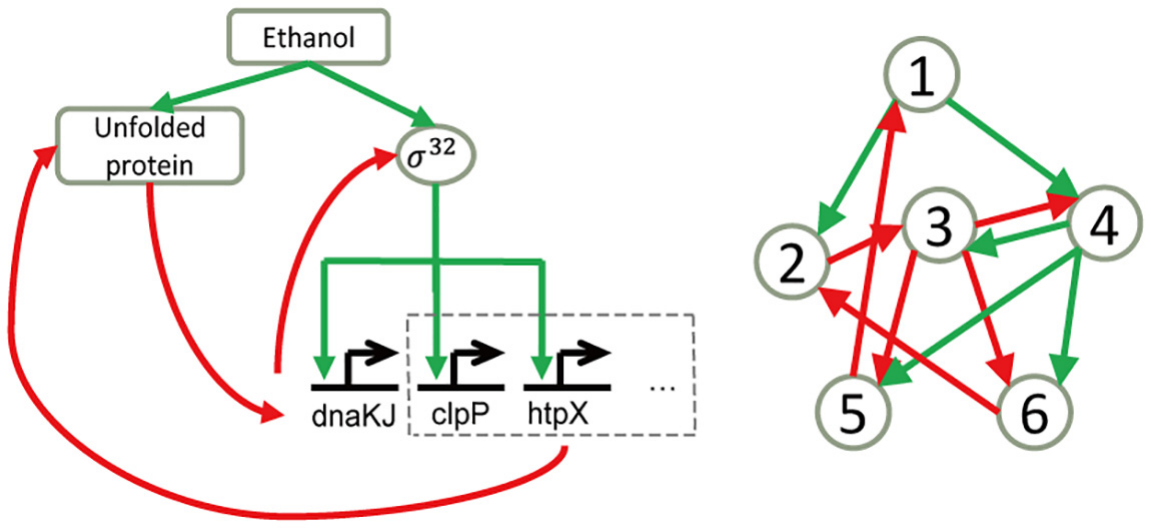
The network for heat shock response in *E*. *coli*. is similar to one of the minimal networks. Left: heat shock response network in *E*. *coli*. Right: one of the 48 minimal networks identified by our algorithm.

The main feature of SOS response is the transit down-regulation of the inhibitor and the activation of functional proteins. Another similar response pathway in *E coli*. is the heat shock response pathway, in which the cell expresses chaperones to refold unfolded proteins induced by high temperature or chemicals such as ethanol. In the response system, the heat shock genes are activated by another sigma factor named σ^32^. σ^32^ is inhibited by chaperone DnaK, which can also bind to unfolded proteins [25]. Upon temperature up-shift, DnaK is driven to form a complex with unfolded proteins, thus releasing σ^32^ from inhibition. Translation of σ^32^ is increased at the same time [26] and heat shock genes are expressed [27], leading to a process as damage is repaired [28]. We found that the heat shock pathway shares a similar structure with networks that are designed to response to DNA damage (Fig 6). Although the two biological networks function in different biological contexts, our finding suggests that response pathways may employ a limited number of strategies to achieve reliable response, and investigating the design principle of one function may hint to the control logic of another.

Our proposed method can largely facilitate design of synthetic circuits that can achieve sequential logic behaviors and can be easily scaled up. Design of functional circuits opens up the possibility of broad application, ranging from biological sensors to medical care [29, 30]. Moreover, illustration of function circuits underlying known biological functions helps us understand the design principles of biological networks [9]. Nevertheless, our method is not without limitations: the performance of our approach may be deteriorated when the dynamics of the network components do not have a significant ON and OFF state. Another issue should be called is that our method does not address the optimization of parameters. In our approach, the networks are ranked according to the volume of the functional parameter space, which is estimated by random sampling of the parameters. However, if possible, the functional parameters should be determined in a wet-lab design. Some of the parameters are not easily adjusted in the experiment, which imposes constraints on the parameter space. In addition, perturbation of several sensitive parameters can dramatically affect the network performance. Thus, further work should be performed to introduce limitations of parameters to contribute to a better design procedure.

## Supporting Information

S1 Fig Budding yeast cell cycle network and its dynamics. (A) The regulatory network of the budding yeast cell cycle network. The nodes represent the signal and the essential proteins. The green lines represent activation and the red lines represent inhibition. (B) Dynamics of cell cycle process in the Boolean network model. (C) Three criteria and their representations in the discrete and continuous model. The first criteria addresses the duration and separation times of the different cell phases. In the second criteria, the final state of the system should return to the G1 state, except for the inactivation of Cln3. The third criterion requires that the dynamics of each node in the ODE model should be in accordance with those in the Boolean trajectory. (D) Example of a successful response in the ODE model.
(TIF)

S2 Fig The distribution of Q value for minimal networks and candidate networks, and the common edges in robust topologies. (A) Distribution of Q value for minimal networks and candidate networks. The minimal networks are represented in red and candidate networks in black. (B) Common edges in robust networks. Black edges represent common edges in all minimal networks. Edges responsible for robust capacity are illustrated in red and green.
(TIF)

S1 Text Performance of our approach on the cell cycle dynamics of budding yeast. (DOC)

## References

[1] Gardner TS, Cantor CR, Collins JJ. Construction of a genetic toggle switch in Escherichia coli. Nature. 2000;403(6767):339–42. . 10659857 10.1038/35002131

[2] Elowitz MB, Leibler S. A synthetic oscillatory network of transcriptional regulators. Nature. 2000;403(6767):335–8. 10.1038/35002125 . 10659856

[3] Danino T, Mondragon-Palomino O, Tsimring L, Hasty J. A synchronized quorum of genetic clocks. Nature. 2010;463(7279):326–30. 10.1038/Nature08753 . 20090747 PMC2838179

[4] Friedland AE, Lu TK, Wang X, Shi D, Church G, Collins JJ. Synthetic Gene Networks That Count. Science. 2009;324(5931):1199–202. 10.1126/science.1172005 19478183 PMC2690711

[5] Wang BJ, Kitney RI, Joly N, Buck M. Engineering modular and orthogonal genetic logic gates for robust digital-like synthetic biology. Nature Communications. 2011;2 Artn 508 10.1038/Ncomms1516 . PMC3207208 22009040

[6] Prindle A, Samayoa P, Razinkov I, Danino T, Tsimring LS, Hasty J. A sensing array of radically coupled genetic 'biopixels'. Nature. 2012;481(7379):39–44. 10.1038/Nature10722 . PMC3259005 22178928

[7] Tabor JJ, Salis HM, Simpson ZB, Chevalier AA, Levskaya A, Marcotte EM, et al A Synthetic Genetic Edge Detection Program. Cell. 2009;137(7):1272–81. 10.1016/j.cell.2009.04.048 . 19563759 PMC2775486

[8] Zhang HQ, Lin M, Shi HD, Ji WY, Huang LW, Zhang XM, et al Programming a Pavlovian-like conditioning circuit in Escherichia coli. Nature Communications. 2014;5 Artn 3102 10.1038/Ncomms4102 . 24434523

[9] Ma WZ, Trusina A, El-Samad H, Lim WA, Tang C. Defining Network Topologies that Can Achieve Biochemical Adaptation. Cell. 2009;138(4):760–73. 10.1016/j.cell.2009.06.013 . 19703401 PMC3068210

[10] Long Y, Qi O, Wang HL. Dose-Response Aligned Circuits in Signaling Systems. PLoS ONE. 2012;7(4). ARTN e34727 10.1371/journal.pone.0034727 . PMC3320644 22496849

[11] Ma WZ, Lai LH, Qi OY, Tang C. Robustness and modular design of the Drosophila segment polarity network. Molecular Systems Biology. 2006;2 Artn 70 10.1038/Msb4100111 . PMC1762089 17170765

[12] Francois P, Hakim V. Design of genetic networks with specified functions by evolution in silico. Proceedings of the National Academy of Sciences of the United States of America. 2004;101(2):580–5. 10.1073/pnas.0304532101 . 14704282 PMC327190

[13] Rodrigo G, Carrera J, Jaramillo A. Genetdes: automatic design of transcriptional networks. Bioinformatics. 2007;23(14):1857–8. 10.1093/bioinformatics/btm237 . 17485427

[14] Dasika MS, Maranas CD. OptCircuit: An optimization based method for computational design of genetic circuits. Bmc Systems Biology. 2008;2 Artn 24 10.1186/1752-0509-2-24 . PMC2324073 18315885

[15] Marchisio MA, Stelling J. Automatic Design of Digital Synthetic Gene Circuits. Plos Computational Biology. 2011;7(2). ARTN e1001083 10.1371/journal.pcbi.1001083 . PMC3048778 21399700

[16] Canton B, Labno A, Endy D. Refinement and standardization of synthetic biological parts and devices. Nature Biotechnology. 2008;26(7):787–93. 10.1038/Nbt1413 . 18612302

[17] Li FT, Long T, Lu Y, Ouyang Q, Tang C. The yeast cell-cycle network is robustly designed. Proceedings of the National Academy of Sciences of the United States of America. 2004;101(14):4781–6. 10.1073/pnas.0305937101 . 15037758 PMC387325

[18] Zhang XM, Shao B, Wu YL, Qi O. A Reverse Engineering Approach to Optimize Experiments for the Construction of Biological Regulatory Networks. PLoS ONE. 2013;8(9). ARTN e75931 10.1371/journal.pone.0075931 . PMC3777925 24069453

[19] Sassanfar M, Roberts JW. Nature of the Sos-Inducing Signal in Escherichia-Coli—the Involvement of DNA-Replication. Journal of Molecular Biology. 1990;212(1):79–96. 10.1016/0022-2836(90)90306-7 . 2108251

[20] Gardner TS, di Bernardo D, Lorenz D, Collins JJ. Inferring genetic networks and identifying compound mode of action via expression profiling. Science. 2003;301(5629):102–5. 10.1126/science.1081900 . 12843395

[21] Wang GY, Du CH, Chen H, Simha R, Rong YW, Xiao Y, et al Process-based network decomposition reveals backbone motif structure. Proceedings of the National Academy of Sciences of the United States of America. 2010;107(23):10478–83. 10.1073/pnas.0914180107 . 20498084 PMC2890799

[22] Shao B, Wu JY, Tian BH, Ouyang Q. Minimum network constraint on reverse engineering to develop biological regulatory networks. Journal of Theoretical Biology. 2014, unpublished. 10.1016/j.jtbi.2015.05.005 25981630

[23] Fisher RJ, Fivash M, Casafinet J, Bladen S, Mcnitt KL. Real-Time Biacore Measurements of Escherichia-Coli Single-Stranded-DNA Binding-Protein to Polydeoxythymidylic Acid Reveal Single State Kinetics with Steric Cooperativity. Faseb J. 1994;8(7):A1271-A. .

[24] Davidich M, Bornholdt S. The transition from differential equations to Boolean networks: A case study in simplifying a regulatory network model. Journal of Theoretical Biology. 2008;255(3):269–77. 10.1016/j.jtbi.2008.07.020 . 18692073

[25] Straus D, Walter W, Gross CA. Dnak, Dnaj, and Grpe Heat-Shock Proteins Negatively Regulate Heat-Shock Gene-Expression by Controlling the Synthesis and Stability of Sigma-32. Gene Dev. 1990;4(12A):2202–9. 10.1101/gad.4.12a.2202 . 2269429

[26] Yuzawa H, Nagai H, Mori H, Yura T. Heat Induction of Sigma(32) Synthesis Mediated by Messenger-Rna Secondary Structure—a Primary Step of the Heat-Shock Response in Escherichia-Coli. Nucleic Acids Research. 1993;21(23):5449–55. 10.1093/nar/21.23.5449 . 7505426 PMC310584

[27] Kanemori M, Mori H, Yura T. Induction of Heat-Shock Proteins by Abnormal Proteins Results from Stabilization and Not Increased Synthesis of Sigma(32) in Escherichia-Coli. Journal of Bacteriology. 1994;176(18):5648–53. . 7916010 10.1128/jb.176.18.5648-5653.1994 PMC196767

[28] Morimoto RI, Kline MP, Bimston DN, Cotto JJ. The heat-shock response: regulation and function of heat-shock proteins and molecular chaperones. Essays in Biochemistry, Vol 32, 1997. 1997;32:17–29. . 9493008

[29] Xiang SL, Fruehauf J, Li CJ. Short hairpin RNA-expressing bacteria elicit RNA interference in mammals. Nature Biotechnology. 2006;24(6):697–702. 10.1038/Nbt.1211 . 16699500

[30] Liu YC, Zeng YY, Liu L, Zhuang CL, Fu X, Huang WR, et al Synthesizing AND gate genetic circuits based on CRISPR-Cas9 for identification of bladder cancer cells. Nature Communications. 2014;5 Artn 5393 10.1038/Ncomms6393 . 25373919

