# Supplementary figures and images for: From Boolean Network Model to Continuous Model Helps in Design of Functional Circuits
Source: PLoS One. 2015 Jun 10;10(6):e0128630. doi: 10.1371/journal.pone.0128630 (PMC4464762; doi:10.1371/journal.pone.0128630)

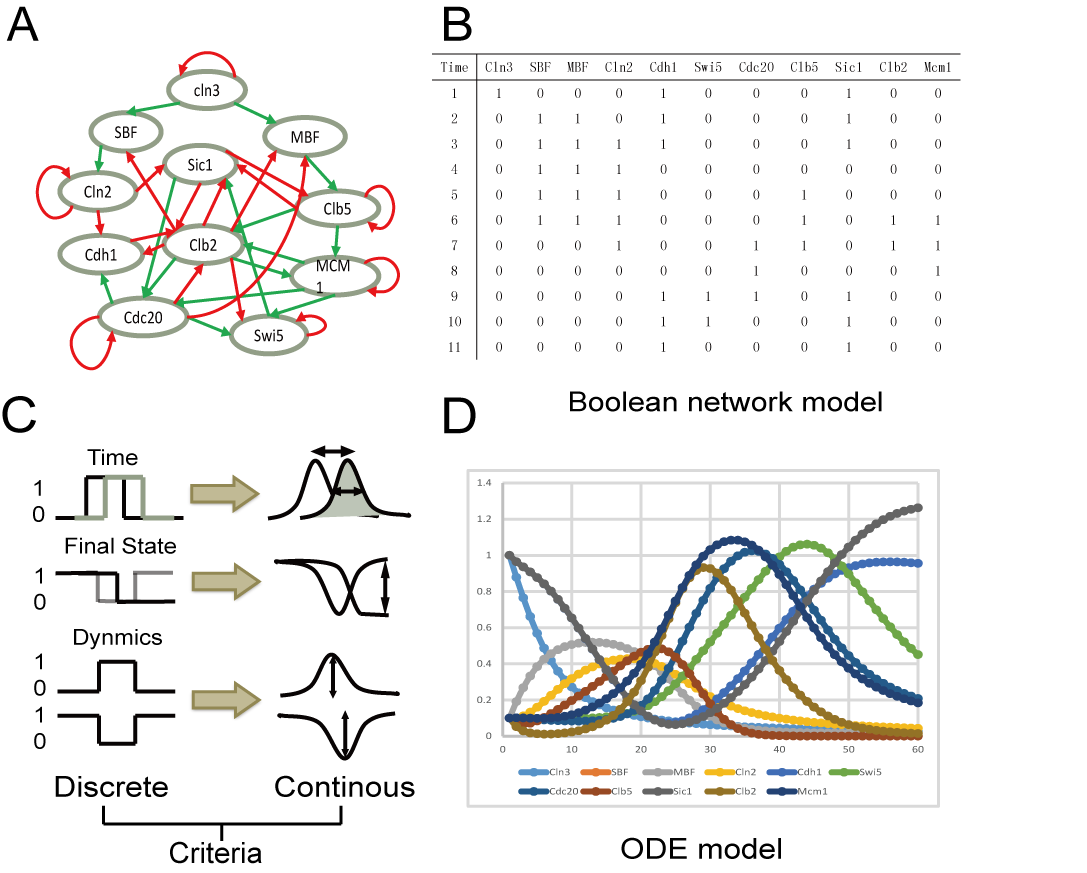

Supplement: S1 Fig — (A) The regulatory network of the budding yeast cell cycle network. The nodes represent the signal and the essential proteins. The green lines represent activation and the red lines represent inhibition. (B) Dynamics of cell cycle process in the Boolean network model. (C) Three criteria and their representations in the discrete and continuous model. The first criteria addresses the duration and separation times of the different cell phases. In the second criteria, the final state of the system should return to the G1 state, except for the inactivation of Cln3. The third criterion requires that the dynamics of each node in the ODE model should be in accordance with those in the Boolean trajectory. (D) Example of a successful response in the ODE model. (TIF) [file pone.0128630.s001.tif]

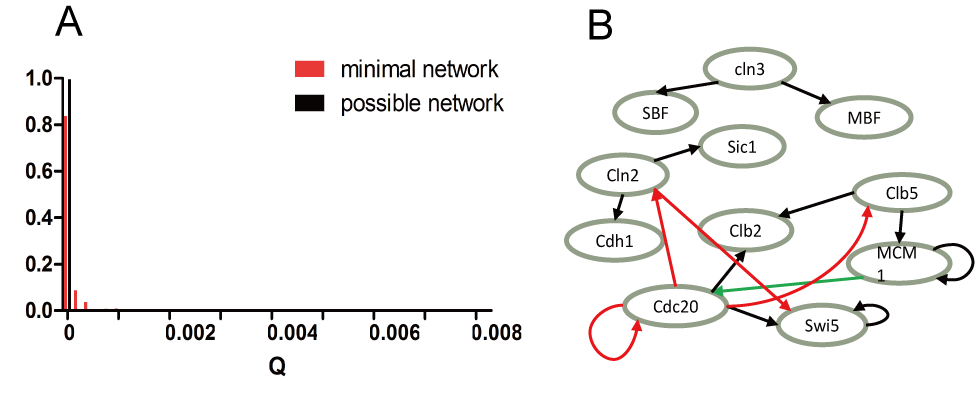

Supplement: S2 Fig — (A) Distribution of Q value for minimal networks and candidate networks. The minimal networks are represented in red and candidate networks in black. (B) Common edges in robust networks. Black edges represent common edges in all minimal networks. Edges responsible for robust capacity are illustrated in red and green. (TIF) [file pone.0128630.s002.tif]
