# Supplementary material for: From Boolean Network Model to Continuous Model Helps in Design of Functional Circuits
Source: PLoS One. 2015 Jun 10;10(6):e0128630. doi: 10.1371/journal.pone.0128630 (PMC4464762; doi:10.1371/journal.pone.0128630)
Supplement: S1 Text — (DOC) [file pone.0128630.s003.doc]

**Cell Cycle Network**

The budding yeast cell cycle network is a well-studied regulatory network that has shown to be functional and structurally stable against perturbations. The cell cycle process consists of four phases: G1 phase for cell growth, S phase for DNA replication, G2 phase for preparation for mitosis, and M phase for mitosis. In addition, several checkpoints exist to ensure that the next event only happens when the former events are completed, e.g., the cell size checkpoint is triggered by the accumulation of cell mass and activates Cln3. Cln3 activates a pair of transcription factors, namely SBF and MBF, which transcriptionally activate the G1/S genes, including Cln2 and Clb5. Next, Sic1 is phosphorylated by Cln2 and later degraded by proteasome, which allows the activation of Clb5. Clb5 initiates the replication of DNA, which is followed by the up-regulation of MCM1. Then, MCM1 stimulates the transcription of G2/M genes, including Clb2 and Swi5. After transcription of Clb2 is stimulated, the cell exits from the cell cycle by activation of Sic1, Cdh1 and Cdc20, and comes back to the stationary G1 state. The entire network is presented in S1 Fig. The Boolean trajectory of the cell cycle process, which was used to obtain all of the candidate networks and minimal networks, is illustrated in S1 Fig. [18].

**Continuous Model Simulation**

We used continuous model simulation to access the dynamic behavior of all candidate networks. The designed network is assumed to be transcriptional network, and its equation is as follows,

$\frac{dx_{i}}{dt}=\left(\sum_{positve} W_{P}a_{j}\frac{x_{j}^{N}+\delta_{ij}}{b_{j}+x_{j}^{N}}\right)\prod_{negative} \frac{1}{c_{j}+W_{N}d_{j}x_{j}^{N}}+0.1-x_{i}$ (S1)

The simulations are conducted the same way as for the SOS response pathway. To calculate the Q value of the networks, we apply similar criteria of dynamic pattern and final states, in addition to restrictions on the key regulators in the cell cycle process. We assume that a successful cell cycle should meet the following conditions (S1 Fig.):

(i) Dynamics patterns of proteins in continuous model simulation must be the same as those in the Boolean trajectory.

(ii) In the final state, only Sic1 and Cdh1 are activated to ensure that the cell remains in stationary state unless there is another round of signals. In other words, Sic1 and Cdh1 should have expression levels larger than 0.1, and other genes should have expression levels lower than 0.1 at the end of simulation.

(iii) Early onset of M phase may result in cell death; thus, we introduce a minimal time period for DNA replication and cell growth. In the simulation, the peak time of Clb2 and Cln2 should be separated by at least 10 (arbitrary unit). The full width at half maximum of Clb2 and Cln2 should be at least 8 (arbitrary unit) at the same time, which ensures a lower boundary of cell phase time.

In addition, we add a constraint that the maximum expression of each gene should be larger than 0.1. These criteria correspond to the main characteristics of the Boolean trajectory, leaving details, such as specific time of activation, unconstrained. We select 1000 candidate networks and 1000 minimal networks to evaluate their performance. A total 10000 sets of parameters are chosen randomly using Latin hypercube sampling to calculate the Q value of each network.

**Results**

For cell cycle dynamics, the Q value distribution of the candidate networks and the minimal networks are shown in S2 Fig. Approximately 1.1% of the candidate networks and 17% of the minimal networks have a Q value above zero, which is reasonable, as the cell cycle dynamics is much more complicated than the SOS response. We also sampled 500 random networks and found that none of these networks have at least one set of functional parameters, indicating that it is unlikely to generate a functional network by random sampling. We can also observe the trend that minimal networks are more robust than candidate networks from the two distributions. The JSD of the distribution for minimal networks and candidate networks is 0.053 with a p-value of 0.0005, showing that the distribution of the Q value of minimal networks is significantly different from that of candidate networks.

We further selected out the most robust topologies (Q > 0.0045) from the minimal networks and determined all the common edges (S2 Fig.). In the topology analysis, all the nodes are also numbered as in the sequence of S1 Fig. Aside from the common edges of all of the candidate networks, these edges may provide the robust capacity of the corresponding networks. Of all the common edges in the robust topologies, inhibition of node 8 by node 7 corresponds to negative feedback from M genes to G1/S genes. Inhibition of node 4 (Cln2) is stimulated via activation of node 7 instead of a self-inhibition loop in the biological network, which may result from the fact that inhibition from other genes may require less time to degrade the gene.

In conclusion, our results in cell cycle dynamics reveal that for distinct functions, transformation from the Boolean network model helps us to select networks that are better capable of exerting target functions, and minimum network constrain further improves the performance of candidate networks.
